# Aquaporin-4 Polymorphisms Are Associated With Cognitive Performance in Parkinson’s Disease

**DOI:** 10.3389/fnagi.2021.740491

**Published:** 2022-03-09

**Authors:** Yi Fang, Shaobing Dai, Chongyao Jin, Xiaoli Si, Luyan Gu, Zhe Song, Ting Gao, Ying Chen, Yaping Yan, Xinzhen Yin, Jiali Pu, Baorong Zhang

**Affiliations:** ^1^Department of Neurology, Second Affiliated Hospital, School of Medicine, Zhejiang University, Hangzhou, China; ^2^Department of Anesthesiology, Women’s Hospital, School of Medicine, Zhejiang University, Hangzhou, China

**Keywords:** Parkinson’s disease, cognitive dysfunction, aquaporin-4, amyloid plaque, REM sleep behavior disorder

## Abstract

**Objective:**

Aquaporin-4 (AQP4) facilitates a sleep-enhanced interstitial brain waste clearance system. This study was conducted to determine the clinical implication of *AQP4* polymorphisms in Parkinson’s disease (PD).

**Methods:**

Three-hundred and eighty-two patients with PD and 180 healthy controls with a mean follow-up time of 66.1 months from the Parkinson’s Progression Marker Initiative study were analyzed. We examined whether *AQP4* SNPs were associated with an altered rate of motor or cognitive decline using linear mixed model and Cox regression. We then investigated whether *AQP4* SNPs were associated with Aβ burden as measured by ^18^F Florbetapir standard uptake values. Furthermore, we examined if *AQP4* SNPs moderated the association between REM sleep behavior disorder (RBD) and CSF biomarkers.

**Results:**

In patients with PD, *AQP4* rs162009 (AA/AG vs. GG) was associated with slower dementia conversion, better performance in letter-number sequencing and symbol digit modalities, lower Aβ deposition in the putamen, anterior cingulum, and frontotemporal areas. In the subgroup of high RBD screening questionnaire score, rs162009 AA/AG had a higher CSF Aβ42 level. rs162009 AA/AG also had better performance in semantic fluency in healthy controls. Besides, rs68006382 (GG/GA vs. AA) was associated with faster progression to mild cognitive impairment, worse performance in letter-number sequencing, semantic fluency, and symbol digit modalities in patients with PD.

**Interpretation:**

Genetic variations of *AQP4* and subsequent alterations of glymphatic efficacy might contribute to an altered rate of cognitive decline in PD. *AQP4* rs162009 is likely a novel genetic prognostic marker of glymphatic function and cognitive decline in PD.

## Introduction

Aquaporin-4 (AQP4) is a water channel that lies at the astrocytic endfeet around perivascular space in the brain (Nagelhus and Ottersen, 2013). AQP4 plays a central role in the glymphatic system, which facilitates exchange between cerebrospinal fluid (CSF) and interstitial fluid, and drains macromolecules from the brain to the periphery (Mestre et al., 2018). Strikingly, the AQP4-facilitated glymphatic system clears interstitial brain waste, including amyloid β (Aβ; Iliff et al., 2012), tau (Harrison et al., 2020), and α-synuclein (Zou et al., 2019) according to animal studies. The association between AQP4 and interstitial waste clearance was further validated by a postmortem study of aging human brains, which revealed a link between reduced perivascular localization of AQP4 and Aβ deposition (Zeppenfeld et al., 2017). In addition, Single Nucleotide Polymorphisms (SNPs) of the *AQP4* gene were associated with brain Aβ uptake on PET and the rate of cognitive decline in the spectrum of Alzheimer’s disease (AD; Burfeind et al., 2017; Chandra et al., 2020).

Parkinson’s disease (PD) is pathologically characterized by intraneuronal α-synuclein accumulation (Jakes et al., 1994), although extracellular α-synuclein is present and might contribute to the seeding of the disease (Lee et al., 2014). In addition, AD pathologies, including extracellular amyloid and tau aggregates, are also found in PD (Robinson et al., 2018). Amyloid deposition as measured by PET was positive in 34% of patients with PD dementia and 5% of patients with PD-mild cognitive impairment (MCI; Petrou et al., 2015). Furthermore, even subthreshold amyloid might contribute to cognitive decline in PD. In patients with PD, lower baseline CSF Aβ42 is associated with a faster rate of cognitive decline (Compta et al., 2013; Alves et al., 2014; Bäckström et al., 2015), worse performance in executive function (Compta et al., 2009; Stav et al., 2015), and delayed memory recall (Hall et al., 2015). Therefore, it is likely that clearing efficacy of synuclein and AD co-pathology are associated with an altered rate of motor or cognitive progression in PD.

Glymphatic fluid transport is known to be sleep-enhanced (Xie et al., 2013) and regulated by circadian rhythms (Hablitz et al., 2020). Disrupted sleep is a potential mechanism accounting for glymphatic dysfunction in PD. REM sleep behavior disorder (RBD) is one of the most characteristic sleep disorders in PD (Xie et al., 2019). Concomitant RBD in patients with PD is associated with faster motor progression, and worse cognitive function (Pagano et al., 2018; Zhang et al., 2020). These patients exhibit elevated synuclein deposition across the brain according to a postmortem study (Postuma et al., 2015). Interestingly, they are also inclined to have lower CSF Aβ42 and higher total tau/Aβ42 levels (Pagano et al., 2018). The elevated synuclein and amyloid burden are potentially attributable to the reduced efficacy of the AQP4-facilitated glymphatic system as a result of sleep disturbance (Bohnen and Hu, 2019).

In this study, using longitudinal data from the Parkinson’s Progression Marker Initiative (PPMI), we tested the hypothesis that *AQP4* SNPs were associated with altered CSF or PET biomarker values and rate of motor or cognitive decline in PD. We also studied if* AQP4* SNPs modulate the association between RBD and CSF biomarkers.

## Materials and Methods

### Study Participants

Major inclusion criteria for the PD cohort in the PPMI were as follows: (1) drug naïve; (2) diagnosed with PD within the past 2 years; (3) Hoehn and Yahr stage 1 or 2 at baseline; (4) age 30 years or older; and (5) striatal dopaminergic dysfunction on SPECT.

To exclude the potential confounding effect of race and ethnicity, non-Hispanic Caucasian patients and healthy controls (HC) were included in our analysis. Causes of exclusion include: (1) no CSF biomarker study result; and (2) no whole-genome sequencing data. Overall, 382 patients and 180 HCs were included, their demographic information and clinical characteristics are presented in Table 1.

**Table 1:** Demographic information and clinical characteristics of patients and healthy controls.

	Patients	Healthy controls
Age, y	61.83 ± 9.50	61.42 ± 10.63
Sex, male, n (%)	252 (66.0%)	117 (65.0%)
Years of education	15.56 ± 2.93	16.18 ± 2.91
*APOE*ε4 carriage	0.28 ± 0.50	0.28 ± 0.51
Baseline MDS-UPDRS I	5.43 ± 4.14	2.84 ± 2.80
Baseline MDS-UPDRS II	5.99 ± 4.24	0.40 ± 0.95
Baseline MDS-UPDRS III	20.96 ± 8.92	1.71 ± 2.16
Baseline MoCA	27.24 ± 0.11	28.23 ± 1.11
Baseline BJLOT	12.14 ± 2.92	12.50 ± 2.75
Baseline HVLT-total recall	45.84 ± 10.61	49.72 ± 9.78
Baseline LNS	11.59 ± 2.63	11.78 ± 2.76
Baseline SFT-T score	51.19 ± 9.86	52.71 ± 10.27
Baseline SDMT-T score	45.00 ± 9.07	50.68 ± 10.10
Baseline ESS	5.80 ± 3.47	5.63 ± 3.36
Baseline RBDSQ	4.53 ± 2.87	2.78 ± 2.29

*MDS-UPDRS, Movement Disorder Society-sponsored revision of the Unified Parkinson’s Disease Rating Scale; MoCA, Montreal Cognitive Assessment; BJLOT, Benton Judgment of line orientation test; HVLT, Hopkins verbal learning test; LNS, Letter-number sequencing; SFT, Semantic fluency test; SDMT, Symbol digit modalities; ESS, Epworth Sleepiness scale; RBDSQ, REM sleep behavior disorder screening questionnaire*.

### Standard Protocol Approvals, Registrations, and Patient Consents

The study was approved by the institutional review board at each PPMI site. All patients signed an informed consent form before their participation in the PPMI study.

### Clinical Assessment

Cognitive function was evaluated on a yearly basis. Cognitive tests included the Montreal Cognitive Assessment for global cognition, the Hopkins Verbal Learning Test (HVLT) for memory, the Benton Judgment of Line Orientation Test (BJLOT) for visuospatial perception, the Semantic Fluency Test (SFT) for executive function, the Letter-Number Sequencing (LNS) and the Symbol Digit Modality Test (SDMT) for working memory and attention-processing speed. Cognitive categorization of normal, MCI, and dementia was performed by investigators at each site in accordance with MDS criteria (Emre et al., 2007; Litvan et al., 2012).

Motor symptoms were evaluated at baseline, 3, 6, 9, 12, 18, 24, 30, 36, 42, 48, 54, 60, 72, 84, 96, and 108 months. We analyzed Hoehn and Yahr stage that was rated during the off condition (levodopa/dopaminergic agonist withheld for at least 6 h prior to the visit).

RBD was evaluated with an RBD screening questionnaire (RBDSQ). The optimal cutoff value for probable RBD in PD is 6 (Stiasny-Kolster et al., 2007; Nomura et al., 2011). RBD symptoms are time-varying. To study if *AQP4* modulates the association between RBD and CSF biomarker, we stratified patients into subgroups by their averaged RBDSQ score in the first 3 years, as CSF samples were collected from baseline to the 3rd year. Overall, there are four expected visits for RBDSQ evaluation during this time frame (baseline, 1st, 2nd, and 3rd-year). Patients who were absent for two or more of these scheduled visits were excluded due to insufficient available data to characterize RBD symptoms within this time frame (*N* = 40). Then we stratified patients into a high RBDSQ group if averaged 3-year RBDSQ lies in the top one-third (RBDSQ ≥ 5.5, *N* = 117), and a low RBDSQ group if averaged 3-year RBDSQ lies in the least one-third (RBDSQ ≤ 3, *N* = 113).

### Genotyping and SNP Pruning

**Figure 1:**
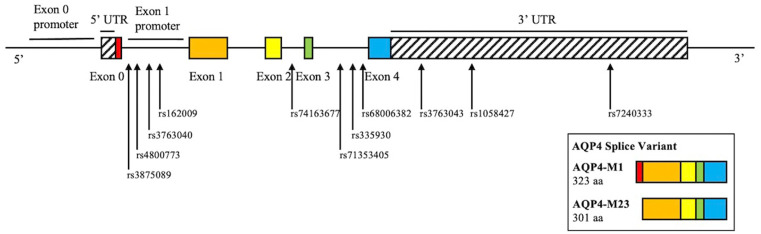
Schematic illustration of the investigated SNPs in the *AQP4* gene. There are two isoforms of AQP4, M1, and M23. M1 consists of exon 0–4, M23 consists of exon 1–4. rs162009 locates in between exon 0 and 1, which presumably acts as the promoter of the M23 isoform.

Whole-genome sequencing data were downloaded from the PPMI database. SNP pruning was undertaken using PLINK (Purcell et al., 2007). Genetic variants of *AQP4* underwent quality control procedures. Specifically, SNPs that were not in Hardy-Weinberg equilibrium (*p* < 0.05), had a minor allele frequency of <5% were removed. Linkage disequilibrium-based SNP pruning was performed to reduce statistical redundancy and maintain coverage of the *AQP4* gene. SNP pruning parameters were as follows: window size 10, increment 5, and variance inflation factor 2. Two SNP pairs left after pruning were in high linkage disequilibrium (rs1058427 and rs12968026: *r*^2^ = 0.96; rs335930 and rs455671: *r*^2^ = 0.86). Therefore, rs12968026 and rs455671 were removed from further statistical analysis, leaving 11 SNPs. The information of these SNPs was displayed in Table 2, Figure 1, and Supplementary Figure 1.

A dominant model was used for all *AQP4* SNPs.

**Table 2:** *AQP4* SNPs being investigated in the current study.

	Annotation	Position (GRCh38)	Minor allele frequency	Previously reported associations
rs7240333 C/T	3’-UTR	18:26852848	0.098	
rs1058427 G/T	3’-UTR	18:26855131	0.115	Risk of cerebral edema (Appelboom et al., 2015)
rs3763043 C/T	3’-UTR	18:26855854	0.318	Risk of intracerebral hemorrhage (Dardiotis et al., 2019)
				and schizophrenia (Wu et al., 2020)
rs68006382 A/G	intron	18:26856555	0.205	
rs335930 A/C	intron	18:26856961	0.228	
rs71353405 C/T	intron	18:26857429	0.061	
rs74163677 G/A	intron	18:26861697	0.055	
rs162009 G/A	M23 promoter	18:26864250	0.344	
rs3763040 G/A	M23 promoter	18:26864410	0.192	Rate of cognitive decline in AD (Burfeind et al., 2017)
rs4800773 G/A	M23 promoter	18:26865017	0.356	
rs3875089 T/C	M23 promoter	18:26865469	0.149	Rate of cognitive decline in AD (Burfeind et al., 2017)

### Aβ Retention by PET

Overall, 36 patients who met the inclusion criteria of the current study underwent a Florbetaben (FBB) PET imaging for *in vivo* imaging of Aβ plaques. We analyzed standard uptake values (SUVs) downloaded from the PPMI dataset. SUV ratios (SUVRs) were calculated using the cerebellar cortex as reference.

### Measurement of CSF Biomarkers

CSF was collected at baseline, 6, 12, 24, and 36 months. Levels of CSF Aβ42, total tau (*t*-tau), and phosphorylated tau (*p*-tau) at threonine 181 position were measured using Elecsys electrochemiluminescence immunoassays on the cobas e 601 analysis platform. CSF α-synuclein concentrations were analyzed using commercially available enzyme-linked immunosorbent assay kits. These values were downloaded from the PPMI database.

### Statistical Analysis

Clinical and biomarker data were downloaded from the PPMI database in December 2020. Statistical analysis was performed using SPSS (IBM Corp, Armonk NY, USA). Graphs were plotted using GraphPad (San Diego, CA, USA).

For longitudinal cognitive test score analysis, mixed models with random slope and intercept and unstructured covariance were used. Fixed effects include age at baseline, sex, baseline MoCA score, years of education, Apolipoprotein-E (*APOE*) ε4 carriage, and *AQP4* SNP of interest. The *AQP4* SNP by time interaction effect was also included as a fixed effect in separate models. Time was treated as a continuous variable. The patient number was listed as a random effect.

Cox proportional hazards regressions were performed to study if *AQP4* polymorphisms were associated with the rate of motor and cognitive deterioration. We defined events separately as: (1) progression to MCI for the first time; (2) progression to dementia for the first time; and (3) progression of Hoehn and Yahr stage for the first time. Age, sex, *APOE* ε4 carriage, years of education, and baseline MoCA score were covaried for conversion to MCI or dementia. Age, sex, baseline MDS-UPDRS-III, and levodopa equivalent dose were covaried for motor progression. Kaplan-Meier curves were plotted, and log-rank test results were presented.

*APOE* ε4 (Mata et al., 2014), glucocerebrosidase (*GBA*) variants (Alcalay et al., 2012), and Catechol-O-methyltransferase (*COMT*) Val^158^Met (rs4680; Egan et al., 2001; Williams-Gray et al., 2009) are previously established genetic markers of cognition in PD. *COMT* rs4680 data was extracted from whole-genome sequencing data. *GBA* variant summary from across different sequencing platforms was directly downloaded. Their distribution on *AQP4* SNPs was tested using the Chi-Square test.

The association between *AQP4* SNPs and SUVRs was examined using linear regression. Covariates included age, sex, *APOE* ε4 carriage, and disease duration at the time of PET scan.

CSF biomarkers (α-synuclein, Aβ, *t*-tau, *p*-tau) did not follow a normal distribution and were, therefore, log10 transformed in the statistical analysis. To determine whether CSF biomarkers differed with respect to *AQP4* polymorphisms across time, linear mixed models with random intercept were used. Fixed effects included age at baseline, sex, time, and *AQP4* SNP of interest. For CSF Aβ42, *APOE* ε4 carriage was also covaried. To determine whether *AQP4* SNPs moderate the association between RBD and CSF biomarkers, the RBDSQ subgroup by *AQP4* SNP interaction effect was tested.

To correct for multiple comparisons, the Benjamini-Hochberg procedure with a false discovery rate at 0.10 was used given the exploratory nature of this study. *p* < 0.05 was used as the threshold for statistical significance.

## Result

### *AQP4* SNPs and Conversion to MCI and Dementia

We did not observe a significant association between *AQP4* SNPs and motor progression as reflected by MDS-UPDRS-III score increment in mixed model and Hoehn and Yahr stage progression in Cox regression.

**Figure 2:**
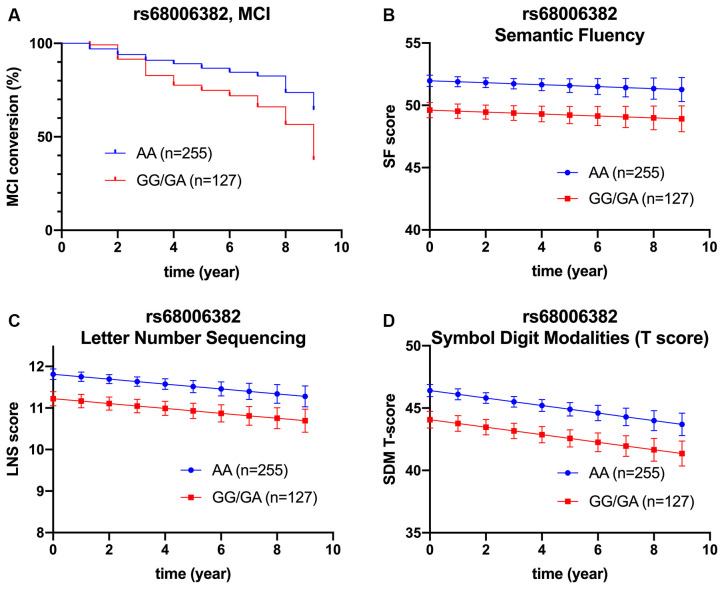
Association between rs68006382 and cognition. rs68006382 (GG/GA vs. AA) had faster conversion to MCI (HR = 1.973, 95% CI = 1.246–3.123, *p =* 0.004) **(A)**. rs68006382 GG/GA genotype also had worse performance in semantic fluency test (*β* = −2.357, SE = 0.691, *p =* 0.001) **(B)**, letter-number sequencing (*β* = −0.587, SE = 0.193, *p =* 0.003) **(C)**, and symbol digit modalities test (*β* = −2.339, SE = 0.193, *p =* 0.002) **(D)**. Covariates for cox regression and linear mixed model included age, sex, *APOE* ε4 carriage, years of education, and baseline MoCA score. Lines in Panels **(B–D)** represent the estimated marginal mean from the mixed model. MCI, mild cognitive impairment.

**Figure 3:**
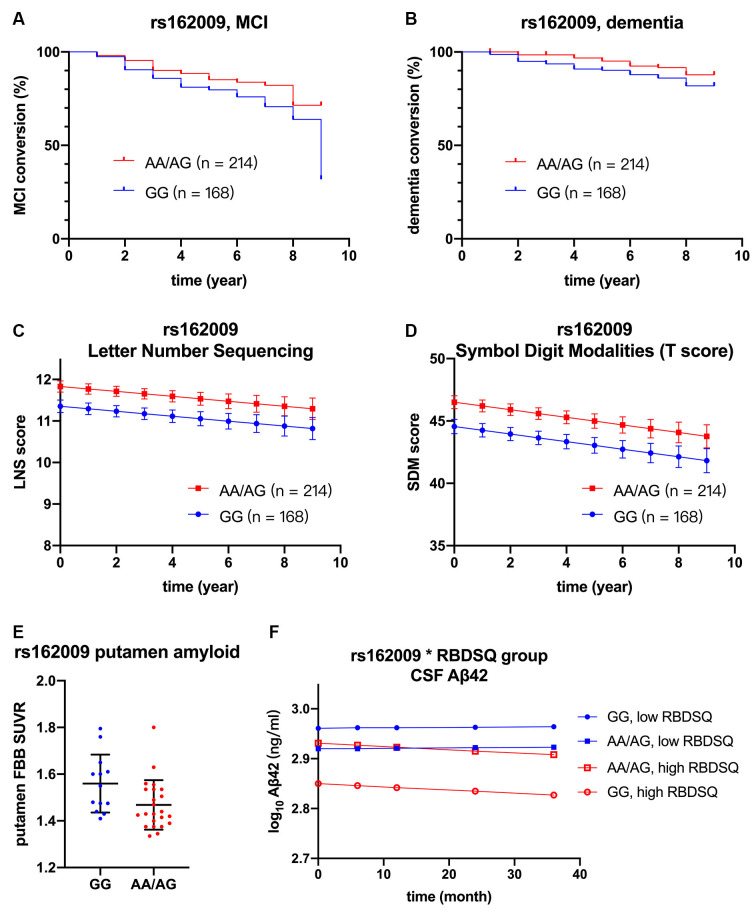
Association between rs162009, cognition, and biomarkers. rs162009 (AA/AG vs. GG) had slower conversion to MCI (HR = 0.646, 95% CI = 0.409–1.019, *p* = 0.060) **(A)** and dementia (HR = 0.473, 95% CI = 0.234–0.957, *p* = 0.037)** (B)**, better performance in letter-number sequencing (*β* = 0.477, SE = 0.183, *p* = 0.009) **(C)**, and symbol digit modalities test (*β* = 2.401, SE = 1.953, *p* = 0.006) **(D)**. rs162009 AA/AG genotype also had lower putamen FBB SUVRs (*β* = −0.105, SE = 0.039, *p* = 0.012) **(E)**. In the subgroup of patients with high RBDSQ score (averaged score during the first 3 year ≥ 5.5, *N* = 117), patients with rs162009 AA/AG genotype had higher CSF Aβ42 levels (β = 0.081, SE = 0.033, *p* = 0.017), indicating its protective effect on Aβ deposition **(F)**. Covariates for cox regression and linear mixed model included age, sex, *APOE* ε4 carriage, years of education, and baseline MoCA score. Lines in Panels **(C,D,F)** represent the estimated marginal mean from the mixed model. FBB, ^18^F Florbetaben; MCI, mild cognitive impairment; RBDSQ, Rapid Eye Movement Sleep Behavior Disorder Screening Questionnaire; SUVRs, standard uptake value ratios.

However, after adjusting for age, sex, *APOE* ε4 carriage, and baseline MoCA score, minor allele carrier status of rs68006382 was associated with accelerated conversion to MCI (HR = 1.973, 95% CI = 1.246–3.123, *p* = 0.004; Figure 2A), but not dementia (HR = 1.512, 95% CI = 0.753–3.034, *p* = 0.245). In addition, minor allele carrier status of rs162009 was associated with slower conversion to MCI (HR = 0.646, 95% CI = 0.409–1.019, *p* = 0.060), and dementia (HR = 0.473, 95% CI = 0.234–0.956, *p* = 0.037), although these effects were marginal (Figures 3A,B).

### *AQP4* SNPs and Performance in Cognitive Subdomains

The association between *AQP4* SNPs and cognitive subdomains is presented in Table 3.

Briefly, minor allele carrier status of rs68006382 was associated with worse performance in executive function, working memory, and attention (SFT: *p* = 0.001; LNS: *p* = 0.003; SDMT: *p* = 0.002; Figures 2B–D).

Minor allele carrier status of rs162009, on the other hand, was protective, with better performance in working memory and attention (LNS: *p* = 0.009; SDMT: *p* = 0.006; Figures 3C,D).

**Table 3:** *AQP4* SNPs and cognition in patients with PD.

	Hazard ratio (95% CI)		Cognitive subdomains (β estimate)				
	MCI	Dementia	BJLOT	HVLT	LNS	SF	SDMT
rs7240333	0.677 (0.355, 1.293)	0.556 (0.194, 1.595)	0.213	1.626	0.308	0.171	0.367
rs1058427	0.668 (0.347, 1.285)	0.564 (0.195, 1.637)	0.038	0.035	0.280	0.457	0.950
rs3763043	1.435 (0.899, 2.292)	1.295 (0.643, 2.606)	−0.089	−0.161	−0.068	**−1.931** ^a^	−0.981
rs68006382	**1.973 (1.246, 3.123)**	1.512 (0.753, 3.034)	−0.229	−1.162	**−0.587** ^a^	**−2.357** ^a^	**−2.339** ^a^
rs335930	0.932 (0.584, 1.485)	0.748 (0.366, 1.530)	−0.178	−0.017	−0.009	−0.387	0.235
rs71353405	0.971 (0.417, 2.258)	0.737 (0.174, 3.120)	−0.063	−1.085	0.150	0.499	−0.243
rs74163677	0.737 (0.365, 1.489)	0.529 (0.160, 1.750)	0.056	0.154	−0.126	−0.964	−0.600
rs162009	0.646 (0.409, 1.019)	**0.473 (0.234, 0.957)**	−0.129	0.583	**0.477** ^a^	1.108	**1.953** ^a^
rs3763040	0.784 (0.481, 1.277)	0.735 (0.348, 1.553)	**0.465** ^a^	1.146	0.333	0.768	0.950
rs4800773	0.896 (0.560, 1.431)	1.034 (0.506, 2.112)	0.141	0.183	0.276	−0.388	−0.486
rs3875089	0.858 (0.490, 1.503)	1.001 (0.440, 2.279)	−0.026	−0.366	0.061	−0.537	0.049

*^a^Significant after correction of multiple comparisons. Covariates include age, sex, *APOE* ɛ4 carriage, years of education, and baseline MoCA score. BJLOT, Benton Judgment of line orientation test; HVLT, Hopkins verbal learning test (total recall); LNS, Letter-number sequencing; SFT, Semantic fluency test; SDMT, Symbol digit modalities test*. Bold values are statistically significant.

In addition, minor allele carrier status of rs3763043 and rs3763040 were associated with worse executive function (SFT: *p* = 0.003) and better visuospatial function (BJLOT: *p* = 0.007), respectively. Minor allele carrier of rs7240333 was associated with better memory (HVLT: *p* = 0.047), which was not significant after correction for multiple comparisons.

There was no significant SNP by time interaction effect in any of the tests. Notably, there was no significant main effect of time in HVLT (*p* = 0.457), LNS (*p* = 0.068), and SFT (*p* = 0.518), indicating the absence of decreasing scores in these subdomains over time. This was likely attributable to the high education level in the PPMI cohort, which amounts to an average of 15.56 years of education. Patients with a higher level of education are likely to have higher cognitive reserve and therefore slower cognitive decline in PD (Meng and D’Arcy, 2012; Hindle et al., 2014).

### Association Between *AQP4* and *APOE* ε4, *COMT* Val^158^Met, *GBA* Variants

Chi-Square test indicated that the distribution of *COMT* variants on rs3763043 minor allele carriers and non-carriers was statistically different (*p* = 0.027), indicating a potential confounding effect of *COMT* variants on the observed association between rs3763043 and SFT. The distribution of *APOE* ε4, *COMT* Val^158^Met, *GBA* variants on other *AQP* SNPs minor allele carrier vs. non-carrier was not significant (Supplementary Table 1).

### *AQP4* SNPs and ^18^F Florbetapir SUVR

Of the 36 patients who underwent ^18^F Florbetapir imaging, six (16.7%) were amyloid positive, if a cut-off of 1.43 for composite SUVR was used (Bullich et al., 2017; Fiorenzato et al., 2018). The average disease duration at the time of PET imaging was 4.73 ± 1.71 years. There was no statistically significant association between SNPs and composite SUVRs, although rs162009 was marginally associated lower composite SUVRs (*β* = −0.076, SE = 0.044, *p* = 0.094).

Given the observed association between minor allele carriers of rs162009 and more preserved cognitive performance, we explored its association with SUVRs in cortical and subcortical regions of interest (Table 4). There were decreased SUVRs in putamen (*p* = 0.012) and anterior cingulum (*p* = 0.048) even with a limited sample size (*n* = 36; Figure 3E). In addition, minor allele carriers of rs162009 were marginally associated with lower amyloid burden in the temporal (*p* = 0.070) and frontal (*p* = 0.072) cortex.

**Table 4:** Association between rs162009 and ^18^F Florbetapir SUVRs.

Regions of interest	β	SE	*p*	Adjusted *r*^2^
putamen	−0.105	0.039	**0.012**	0.159
Anterior cingulum	−0.122	0.059	**0.048**	0.066
orbitofrontal	−0.084	0.042	0.056	0.077
Mesial temporal cortex	−0.065	0.034	0.068	0.046
Lateral temporal cortex	−0.067	0.036	0.070	0.082
Temporal cortex	−0.066	0.035	0.070	0.069
Frontal cortex	−0.084	0.045	0.072	0.102
Parietal cortex	−0.081	0.048	0.102	0.095
Rectus	−0.084	0.050	0.104	0.046
Occipital cortex	−0.062	0.041	0.139	0.018
Posterior cingulum	−0.078	0.066	0.245	−0.002
Thalamus	−0.040	0.052	0.449	−0.037
Caudate	−0.038	0.055	0.494	0.021
Pons	0.023	0.071	0.746	−0.132

*FBB, ^18^F Florbetaben; SUVRs, standard uptake value ratios*. Bold values are statistically significant.

### The Interaction Between *AQP4* SNPs, Sleep Disturbance, and CSF Biomarkers

There was no significant association between *AQP4* SNPs and CSF biomarkers in the whole cohort. Then we examined the association between RBD and CSF biomarkers. Our analysis concords with a previous study that RBD is associated with lower CSF Aβ42 using the same PPMI database (Pagano et al., 2018). There was no such association between RBD and α-synuclein or tau. Given the proposed role of *AQP4* in solute drainage during sleep, we examined if *AQP4* SNPs modulate this association. After controlling for *APOE* ε4 carriage, age, sex and time, minor allele of rs162009 was associated with higher CSF Aβ42 level in the high RBDSQ subgroup (*β* = 0.081, SE = 0.033, *p* = 0.017), but not in the low RBDSQ subgroup (subgroup by SNP interaction effect: *β* = −0.056, SE = 0.023, *p* = 0.014; Figure 3F).

### *AQP4* SNPs and Cognitive Performance in Healthy Controls

The association between *AQP4* SNP and cognitive scales was demonstrated in Table 5. Notably, minor allele carrier status of rs162009 was associated with higher semantic fluency test performance (*β* = 2.209, SE = 0.913, *p* = 0.016), validating the association between rs162009 and cognitive performance observed in patients with PD.

**Table 5:** *AQP4* SNPs and cognition in healthy controls.

	BJLOT		HVLT		LNS		SF		SDMT	
	β	*p*	β	*p*	β	*p*	β	*p*	β	*p*
rs7240333	0.063	0.819	−0.451	0.676	−0.464	0.091	0.502	0.636	−0.961	0.379
rs1058427	0.046	0.872	−1.781	0.108	0.235	0.406	0.810	0.458	0.317	0.778
rs3763043	0.217	0.380	2.000	**0.038**	−0.025	0.921	−0.006	0.995	−0.135	0.891
rs68006382	0.150	0.543	2.954	**0.002** ^a^	0.305	0.216	−0.119	0.900	−0.247	0.801
rs335930	0.178	0.473	−0.519	0.593	−0.093	0.708	0.903	0.345	−0.380	0.701
rs71353405	−0.278	0.441	−0.118	0.933	−0.320	0.377	0.578	0.678	1.231	0.391
rs74163677	0.702	0.122	−4.060	**0.022**	−0.510	0.262	−2.640	0.130	0.737	0.683
rs162009	0.241	0.315	0.087	0.926	0.162	0.499	2.209	**0.016**	0.895	0.348
rs3763040	−0.022	0.929	−0.847	0.379	−0.280	0.255	−0.269	0.777	−1.977	**0.042**
rs4800773	−0.003	0.991	−1.654	0.091	0.000	0.999	0.329	0.733	−1.382	0.164
rs3875089	0.152	0.556	−1.246	0.216	0.126	0.624	1.138	0.251	1.017	0.320

*^a^Significant after correction of multiple comparisons. BJLOT, Benton Judgment of line orientation test; HVLT, Hopkins verbal learning test (total recall); LNS, Letter-number sequencing; SFT, Semantic fluency test; SDMT, Symbol digit modalities test*. Bold values are statistically significant.

In addition, multiple *AQP4* SNPs (rs3763043, rs68006382, and rs74163677) were associated with HVLT performance. However, these associations were likely attributable to the fact that AQP4 plays a role in synaptic plasticity and therefore learning and memory (Hubbard et al., 2018; Woo et al., 2018).

During follow-up, 6/180 (3.33%) and 1/180 (0.56%) HCs developed MCI and dementia, respectively. Therefore, we did not perform Cox regressions in HCs.

Besides, there was no significant distribution difference of *AQP4* SNPs in PD and HCs.

## Discussion

Given the previously established association between *AQP4* and cognitive performance in the spectrum of AD, we explored this association in a cohort of patients with PD. We found that rs162009 was associated with slower dementia conversion, better performance in LNS and SDMT, and lower amyloid burden in the putamen and anterior cingulum. This variant was also associated with higher CSF Aβ in the subgroup with a high RBDSQ score. Besides, rs68006382 was associated with faster MCI conversion and worse performance in LNS, SFT, and SDMT.

The implication of rs162009 AA/AG genotype was more compelling as this variant was associated with both better cognitive performance and lower amyloid deposition in multiple regions. In addition, its protective effect on cognition was also seen in HCs. One of the potential mechanisms to back up these clinical findings is that rs162009 likely increases the transcription of AQP4-M23 isoform and improves the glymphatic fluid circulation. There are two AQP4 splice variants, the M1 and M23. The M1 variant tends to be evenly distributed across the astrocytic membrane, while the M23 variant primarily locates at the astrocytic endfeet (Smith et al., 2014). rs162009 locates between exon 0 and exon 1 of the *AQP4* gene (Figure 1), and putatively acts as a promoter for the AQP4-M23 splice variant, which potentially facilitates the the glymphatic fluid circulation (Iliff et al., 2012). However, this was primarily speculative, more mechanistic study is needed to explore the physiological effect of *AQP4* SNPs.

The current findings add weight to previous observations that even subthreshold amyloid contributes to cognitive decline in non-demented PD. Interestingly, we observed a link between rs162009 and decreased putamen amyloid deposition. Increased striatal amyloid deposition was a prominent feature in PD-dementia autopsy (Kalaitzakis et al., 2008; Hepp et al., 2016). PD patients with combined amyloid pathology in cortex and striatum were associated with worse cognition than those with cortical amyloid pathology alone (Shah et al., 2016). In line with AD, it has been proposed that amyloid deposits in the neocortex first and striatum later (Shah et al., 2016). Therefore, the association between rs162009 and putamen SUVR might correspond to the slowed dementia conversion. The lower amyloid burden in the anterior cingulum (Petersen and Posner, 2012) and frontotemporal area (Randolph et al., 1993; Forn et al., 2009) in rs162009 minor allele carriers might correspond to better attention and working memory performance in these patients. However, AQP4 might modulate cognition in PD through other mechanisms such as synaptic plasticity (Hubbard et al., 2018; Woo et al., 2018), and neuroimmunological regulation (Ikeshima-Kataoka, 2016; Tamtaji et al., 2019).

The association between rs162009 and CSF Aβ42 in the subgroup of high RBDSQ score provides a novel insight into the interaction between RBD and AQP4-facilitated glymphatic clearance. Although glymphatic efficacy peaks during slow-wave sleep (Hablitz et al., 2019), changes in the EEG signature of non-REM sleep have been observed in patients with RBD (Sunwoo et al., 2020). Therefore, it is reasonable to assume that RBD reduces glymphatic capacity. In patients with high RBDSQ scores, disrupted sleep architecture likely reduced the clearing capacity of the glymphatic system, which is attributable to the *AQP4* polymorphisms. The absence of significant association in the low RBDSQ subgroup likely reflected a situation where the glymphatic capacity exceeds the clearing need due to intact sleep. This finding provides indirect evidence that amyloid deposition as a result of disrupted glymphatic clearance might contribute to cognitive decline in RBD. This finding also highlights the prospects of targeting amyloid with immunotherapy in a subgroup of patients with PD (for instance, RBD and non-carriers of rs162009 minor allele).

We did not observe a significant association between *AQP4* and motor progression or CSF α-synuclein. This was likely because α-synuclein was primarily intracellular, the need for interstitial clearance might be low when compared with extracellular Aβ. Although significant α-synuclein aggregation was reported in *Aqp4* knockout mice (Xue et al., 2019), single nucleotide polymorphisms in humans likely exert a much smaller effect than genetic knockout.

The major limitations of the current study are as follows: (1) Significant associations observed in the current study are likely to be false positive. More work in other cohorts is needed to verify the effect of *AQP4* SNPs on PD. (2) We observed a marginally significant effect of rs162009 on FBB SUVRs in a limited number of patients (*n* = 36). Future studies to validate the finding by expanding the patient sample size would be necessary. Besides, such a small number of patients who underwent FBB-PET would limit the statistical power to detect *AQP4* variants that are potentially associated with FBB SUV, especially for those SNPs that have low minor allele frequency. (3) Caution must be taken when interpreting the interaction effect between rs162009 and RBDSQ subgroup. It is also likely that abnormal neuronal activation as a result of sleep disturbance causes elevated production and release of Aβ (Ovsepian and O’Leary, 2016; Lucey, 2020). As Aβ measured by CSF or PET represents the balance of production and clearance, future studies to measure glymphatic activity *in vivo* would be necessary. (4) Measuring RBD symptoms with RBDSQ is prone to subjectivity (Halsband et al., 2018). More studies are warranted to validate and explore the effect of various types of sleep disturbance with objective sleep assessment equipment such as polysomnography and actigraphy.

## Conclusions

Genetic variations of *AQP4* likely alter the glymphatic clearance of Aβ in the brain and subsequently the rate of cognitive decline in PD. *AQP4* rs162009 is likely a novel prognostic marker of cognitive decline in PD. Our findings also provide indirect evidence that in PD, AQP4-facilitated clearance of interstitial amyloid might be disrupted in patients with probable REM sleep behavior disorder.

## Data Availability Statement

Publicly available datasets were analyzed in this study. This data can be found here: https://www.ppmi-info.org/access-data-specimens/download-data.

## Ethics Statement

Ethical approval was not provided for this study on human participants because this study obtained and analyzed data from the PPMI dataset. Ethical approval was obtained at each individual PPMI participating site. The patients/participants provided their written informed consent to participate in the PPMI study.

## Author Contributions

YF, JP, and BZ contributed to the conception and design of the study. YF, SD, and CJ performed the statistical analysis. YF and SD wrote the first draft of the manuscript. All authors contributed to the article and approved the submitted version.

## Conflict of Interest

The authors declare that the research was conducted in the absence of any commercial or financial relationships that could be construed as a potential conflict of interest.

## Publisher’s Note

All claims expressed in this article are solely those of the authors and do not necessarily represent those of their affiliated organizations, or those of the publisher, the editors and the reviewers. Any product that may be evaluated in this article, or claim that may be made by its manufacturer, is not guaranteed or endorsed by the publisher.

## References

[B1] Alcalay R. N. Caccappolo E. Mejia-Santana H. Tang M.-X. Rosado L. Orbe Reilly M.. (2012). Cognitive performance of GBA mutation carriers with early-onset PD. Neurology 78, 1434–1440. 10.1212/WNL.0b013e318253d54b 22442429 PMC3345785

[B2] Alves G. Lange J. Blennow K. Zetterberg H. Andreasson U. Forland M. G.. (2014). CSF Aβ_42_ predicts early-onset dementia in Parkinson disease. Neurology 82, 1784–1790. 10.1212/WNL.0000000000000425 24748671

[B3] Appelboom G. Bruce S. Duren A. Piazza M. Monahan A. Christophe B.. (2015). Aquaporin-4 gene variant independently associated with oedema after intracerebral haemorrhage. Neurol. Res. 37, 657–661. 10.1179/1743132815Y.0000000047 26000774

[B4] Bäckström D. C. Eriksson Domellöf M. Linder J. Olsson B. Öhrfelt A. Trupp M.. (2015). Cerebrospinal fluid patterns and the risk of future dementia in early, incident Parkinson disease. JAMA Neurol. 72, 1175–1182. 10.1001/jamaneurol.2015.1449 26258692

[B5] Bohnen N. I. Hu M. T. M. (2019). Sleep disturbance as potential risk and progression factor for Parkinson’s disease. J. Parkinsons Dis. 9, 603–614. 10.3233/JPD-191627 31227656 PMC6700634

[B6] Bullich S. Seibyl J. Catafau A. M. Jovalekic A. Koglin N. Barthel H.. (2017). Optimized classification of 18F-Florbetaben PET scans as positive and negative using an SUVR quantitative approach and comparison to visual assessment. Neuroimage Clin. 15, 325–332. 10.1016/j.nicl.2017.04.025 28560157 PMC5440277

[B7] Burfeind K. G. Murchison C. F. Westaway S. K. Simon M. J. Erten-Lyons D. Kaye J. A.. (2017). The effects of noncoding aquaporin-4 single-nucleotide polymorphisms on cognition and functional progression of Alzheimer’s disease. Alzheimers Dement. (NY) 3, 348–359. 10.1016/j.trci.2017.05.001 29067342 PMC5651426

[B8] Chandra A. Farrell C. Wilson H. Dervenoulas G. De Natale E. R. Politis M. (2020). Aquaporin-4 polymorphisms predict amyloid burden and clinical outcome in the Alzheimer’s disease spectrum. Neurobiol. Aging 97, 1–9. 10.1016/j.neurobiolaging.2020.06.007 33068891

[B9] Compta Y. Martí M. J. Ibarretxe-Bilbao N. Junqué C. Valldeoriola F. Muñoz E.. (2009). Cerebrospinal tau, phospho-tau and beta-amyloid and neuropsychological functions in Parkinson’s disease: CSF and neuropsychological markers in PD. Mov. Disord. 24, 2203–2210. 10.1002/mds.22594 19795497

[B10] Compta Y. Pereira J. B. Ríos J. Ibarretxe-Bilbao N. Junqué C. Bargalló N.. (2013). Combined dementia-risk biomarkers in Parkinson’s disease: a prospective longitudinal study. Parkinsonism Relat. Disord. 19, 717–724. 10.1016/j.parkreldis.2013.03.009 23643469

[B11] Dardiotis E. Siokas V. Marogianni C. Aloizou A.-M. Sokratous M. Paterakis K.. (2019). AQP4 tag SNPs in patients with intracerebral hemorrhage in greek and polish population. Neurosci. Lett. 696, 156–161. 10.1016/j.neulet.2018.12.025 30578930

[B12] Egan M. F. Goldberg T. E. Kolachana B. S. Callicott J. H. Mazzanti C. M. Straub R. E.. (2001). Effect of COMT Val108/158 Met genotype on frontal lobe function and risk for schizophrenia. Proc. Natl. Acad. Sci. U S A 98, 6917–6922. 10.1073/pnas.111134598 11381111 PMC34453

[B13] Emre M. Aarsland D. Brown R. Burn D. J. Duyckaerts C. Mizuno Y.. (2007). Clinical diagnostic criteria for dementia associated with Parkinson’s disease. Mov. Disord. 22, 1689–1707. 10.1002/mds.21507 17542011

[B14] Fiorenzato E. Biundo R. Cecchin D. Frigo A. C. Kim J. Weis L.. (2018). Brain amyloid contribution to cognitive dysfunction in early-stage Parkinson’s disease: the PPMI dataset. J. Alzheimers Dis. 66, 229–237. 10.3233/JAD-180390 30282359

[B15] Forn C. Belloch V. Bustamante J. C. Garbin G. Parcet-Ibars M. à. Sanjuan A.. (2009). A symbol digit modalities test version suitable for functional MRI studies. Neurosci. Lett. 456, 11–14. 10.1016/j.neulet.2009.03.081 19429124

[B16] Hablitz L. M. Plá V. Giannetto M. Vinitsky H. S. Stæger F. F. Metcalfe T.. (2020). Circadian control of brain glymphatic and lymphatic fluid flow. Nat. Commun. 11:4411. 10.1038/s41467-020-18115-2 32879313 PMC7468152

[B17] Hablitz L. M. Vinitsky H. S. Sun Q. Stæger F. F. Sigurdsson B. Mortensen K. N.. (2019). Increased glymphatic influx is correlated with high EEG delta power and low heart rate in mice under anesthesia. Sci. Adv. 5:eaav5447. 10.1126/sciadv.aav5447 30820460 PMC6392807

[B18] Hall S. Surova Y. Öhrfelt A. Zetterberg H. Lindqvist D. Hansson O. (2015). CSF biomarkers and clinical progression of Parkinson disease. Neurology 84, 57–63. 10.1212/WNL.0000000000001098 25411441 PMC4336091

[B19] Halsband C. Zapf A. Sixel-Döring F. Trenkwalder C. Mollenhauer B. (2018). The REM sleep behavior disorder screening questionnaire is not valid in *de novo* Parkinson’s disease. Mov. Disord. Clin. Pract. 5, 171–176. 10.1002/mdc3.12591 30009211 PMC6033034

[B20] Harrison I. F. Ismail O. Machhada A. Colgan N. Ohene Y. Nahavandi P.. (2020). Impaired glymphatic function and clearance of tau in an Alzheimer’s disease model. Brain 143, 2576–2593. 10.1093/brain/awaa179 32705145 PMC7447521

[B21] Hepp D. H. Vergoossen D. L. E. Huisman E. Lemstra A. W. Bank N. B. Berendse H. W.. (2016). Distribution and load of amyloid-β pathology in Parkinson disease and dementia with lewy bodies. J. Neuropathol. Exp. Neurol. 75, 936–945. 10.1093/jnen/nlw070 27516115

[B22] Hindle J. V. Martyr A. Clare L. (2014). Cognitive reserve in Parkinson’s disease: a systematic review and meta-analysis. Parkinsonism Relat. Disord. 20, 1–7. 10.1016/j.parkreldis.2013.08.010 24034887

[B23] Hubbard J. A. Szu J. I. Binder D. K. (2018). The role of aquaporin-4 in synaptic plasticity, memory and disease. Brain Res. Bull. 136, 118–129. 10.1016/j.brainresbull.2017.02.011 28274814

[B24] Ikeshima-Kataoka H. (2016). Neuroimmunological implications of AQP4 in astrocytes. Int. J. Mol. Sci. 17:1306. 10.3390/ijms17081306 27517922 PMC5000703

[B25] Iliff J. J. Wang M. Liao Y. Plogg B. A. Peng W. Gundersen G. A.. (2012). A paravascular pathway facilitates CSF flow through the brain parenchyma and the clearance of interstitial solutes, including amyloid. Sci. Transl. Med. 4:147ra111. 10.1126/scitranslmed.3003748 22896675 PMC3551275

[B26] Jakes R. Spillantini M. G. Goedert M. (1994). Identification of two distinct synucleins from human brain. FEBS Lett. 345, 27–32. 10.1016/0014-5793(94)00395-5 8194594

[B27] Kalaitzakis M. E. Graeber M. B. Gentleman S. M. Pearce R. K. B. (2008). Striatal β-amyloid deposition in Parkinson disease with dementia. J. Neuropathol. Exp. Neurol. 67, 155–161. 10.1097/NEN.0b013e31816362aa 18219254

[B28] Lee H.-J. Bae E.-J. Lee S.-J. (2014). Extracellular α-synuclein—a novel and crucial factor in Lewy body diseases. Nat. Rev. Neurol. 10, 92–98. 10.1038/nrneurol.2013.275 24468877

[B29] Litvan I. Goldman J. G. Tröster A. I. Schmand B. A. Weintraub D. Petersen R. C.. (2012). Diagnostic criteria for mild cognitive impairment in Parkinson’s disease: movement disorder society task force guidelines: PD-MCI diagnostic criteria. Mov. Disord. 27, 349–356. 10.1002/mds.24893 22275317 PMC3641655

[B30] Lucey B. P. (2020). It’s complicated: the relationship between sleep and Alzheimer’s disease in humans. Neurobiol. Dis. 144:105031. 10.1016/j.nbd.2020.105031 32738506 PMC7484285

[B31] Mata I. F. Leverenz J. B. Weintraub D. Trojanowski J. Q. Hurtig H. I. Van Deerlin V. M.. (2014). APOE, MAPT and SNCA genes and cognitive performance in Parkinson disease. JAMA Neurol. 71:1405. 10.1001/jamaneurol.2014.1455 25178429 PMC4227942

[B32] Meng X. D’Arcy C. (2012). Education and dementia in the context of the cognitive reserve hypothesis: a systematic review with meta-analyses and qualitative analyses. PLoS One 7:e38268. 10.1371/journal.pone.0038268 22675535 PMC3366926

[B33] Mestre H. Hablitz L. M. Xavier A. L. Feng W. Zou W. Pu T.. (2018). Aquaporin-4-dependent glymphatic solute transport in the rodent brain. eLife 7:e40070. 10.7554/eLife.40070 30561329 PMC6307855

[B34] Nagelhus E. A. Ottersen O. P. (2013). Physiological roles of aquaporin-4 in brain. Physiol. Rev. 93, 1543–1562. 10.1152/physrev.00011.2013 24137016 PMC3858210

[B35] Nomura T. Inoue Y. Kagimura T. Uemura Y. Nakashima K. (2011). Utility of the REM sleep behavior disorder screening questionnaire (RBDSQ) in Parkinson’s disease patients. Sleep Med. 12, 711–713. 10.1016/j.sleep.2011.01.015 21700495

[B36] Ovsepian S. V. O’Leary V. B. (2016). Neuronal activity and amyloid plaque pathology: an update. J. Alzheimers Dis. 49, 13–19. 10.3233/JAD-150544 26444792

[B37] Pagano G. De Micco R. Yousaf T. Wilson H. Chandra A. Politis M. (2018). REM behavior disorder predicts motor progression and cognitive decline in Parkinson disease. Neurology 91, e894–e905. 10.1212/WNL.0000000000006134 30089615

[B38] Petersen S. E. Posner M. I. (2012). The attention system of the human brain: 20 years after. Annu. Rev. Neurosci. 35, 73–89. 10.1146/annurev-neuro-062111-150525 22524787 PMC3413263

[B39] Petrou M. Dwamena B. A. Foerster B. R. MacEachern M. P. Bohnen N. I. Müller M. L.. (2015). Amyloid deposition in Parkinson’s disease and cognitive impairment: a systematic review: systematic review: amyloid in Parkinsonian dementia. Mov. Disord. 30, 928–935. 10.1002/mds.26191 25879534 PMC4478091

[B40] Postuma R. B. Gagnon J.-F. Bertrand J.-A. Génier Marchand D. Montplaisir J. Y. (2015). Parkinson risk in idiopathic REM sleep behavior disorder. Neurology 84, 1104–1113. 10.1212/WNL.0000000000001364 25681454 PMC4371408

[B41] Purcell S. Neale B. Todd-Brown K. Thomas L. Ferreira M. A. R. Bender D.. (2007). PLINK: a tool set for whole-genome association and population-based linkage analyses. Am. J. Hum. Genet. 81, 559–575. 10.1086/519795 17701901 PMC1950838

[B42] Randolph C. Braun A. R. Goldberg T. E. Chase T. N. (1993). Semantic fluency in Alzheimer’s, Parkinson’s and Huntington’s disease: dissociation of storage and retrieval failures. Neuropsychology 7, 82–88. 10.1037/0894-4105.7.1.82

[B43] Robinson J. L. Lee E. B. Xie S. X. Rennert L. Suh E. Bredenberg C.. (2018). Neurodegenerative disease concomitant proteinopathies are prevalent, age-related and APOE4-associated. Brain 141, 2181–2193. 10.1093/brain/awy146 29878075 PMC6022546

[B44] Shah N. Frey K. A. Müller L. T. M. Petrou M. Kotagal V. Koeppe R. A.. (2016). Striatal and cortical β-amyloidopathy and cognition in Parkinson’s disease: striatal β-amyloid and cognition in PD. Mov. Disord. 31, 111–117. 10.1002/mds.26369 26380951 PMC4724301

[B45] Smith A. J. Jin B.-J. Ratelade J. Verkman A. S. (2014). Aggregation state determines the localization and function of M1- and M23-aquaporin-4 in astrocytes. J. Cell Biol. 204, 559–573. 10.1083/jcb.201308118 24515349 PMC3926963

[B46] Stav A. L. Aarsland D. Johansen K. K. Hessen E. Auning E. Fladby T. (2015). Amyloid-β and α-synuclein cerebrospinal fluid biomarkers and cognition in early Parkinson’s disease. Parkinsonism Relat. Disord. 21, 758–764. 10.1016/j.parkreldis.2015.04.027 25971633

[B47] Stiasny-Kolster K. Mayer G. Schäfer S. Möller J. C. Heinzel-Gutenbrunner M. Oertel W. H. (2007). The REM sleep behavior disorder screening questionnaire—A new diagnostic instrument. Mov. Disord. 22, 2386–2393. 10.1002/mds.21740 17894337

[B48] Sunwoo J.-S. Cha K. S. Byun J.-I. Jun J.-S. Kim T.-J. Shin J.-W.. (2020). Nonrapid eye movement sleep electroencephalographic oscillations in idiopathic rapid eye movement sleep behavior disorder: a study of sleep spindles and slow oscillations. Sleep 44:zsaa160. 10.1093/sleep/zsaa160 32827438

[B49] Tamtaji O. R. Behnam M. Pourattar M. A. Jafarpour H. Asemi Z. (2019). Aquaporin 4: a key player in Parkinson’s disease. J. Cell. Physiol. 234, 21471–21478. 10.1002/jcp.28871 31127615

[B50] Williams-Gray C. H. Evans J. R. Goris A. Foltynie T. Ban M. Robbins T. W.. (2009). The distinct cognitive syndromes of Parkinson’s disease: 5 year follow-up of the CamPaIGN cohort. Brain 132, 2958–2969. 10.1093/brain/awp245 19812213

[B51] Woo J. Kim J. E. Im J. J. Lee J. Jeong H. S. Park S.. (2018). Astrocytic water channel aquaporin-4 modulates brain plasticity in both mice and humans: a potential gliogenetic mechanism underlying language-associated learning. Mol. Psychiatry 23, 1021–1030. 10.1038/mp.2017.113 29565042

[B52] Wu Y.-F. Sytwu H.-K. Lung F.-W. (2020). Polymorphisms in the human aquaporin 4 gene are associated with schizophrenia in the southern chinese han population: a case-control study. Front. Psychiatry 11:596. 10.3389/fpsyt.2020.00596 32676041 PMC7333661

[B53] Xie F. Gao X. Yang W. Chang Z. Yang X. Wei X.. (2019). Advances in the research of risk factors and prodromal biomarkers of Parkinson’s disease. ACS Chem. Neurosci. 10, 973–990. 10.1021/acschemneuro.8b00520 30590011

[B54] Xie L. Kang H. Xu Q. Chen M. J. Liao Y. Thiyagarajan M.. (2013). Sleep drives metabolite clearance from the adult brain. Science 342, 373–377. 10.1126/science.1241224 24136970 PMC3880190

[B55] Xue X. Zhang W. Zhu J. Chen X. Zhou S. Xu Z.. (2019). Aquaporin-4 deficiency reduces TGF-β1 in mouse midbrains and exacerbates pathology in experimental Parkinson’s disease. J. Cell. Mol. Med. 23, 2568–2582. 10.1111/jcmm.14147 30680924 PMC6433854

[B57] Zeppenfeld D. M. Simon M. Haswell J. D. D’Abreo D. Murchison C. Quinn J. F.. (2017). Association of perivascular localization of aquaporin-4 with cognition and Alzheimer disease in aging brains. JAMA Neurol. 74, 91–99. 10.1001/jamaneurol.2016.4370 27893874

[B58] Zhang F. Niu L. Liu X. Liu Y. Li S. Yu H.. (2020). Rapid eye movement sleep behavior disorder and neurodegenerative diseases: an update. Aging Dis. 11, 315–326. 10.14336/AD.2019.0324 32257544 PMC7069464

[B59] Zou W. Pu T. Feng W. Lu M. Zheng Y. Du R.. (2019). Blocking meningeal lymphatic drainage aggravates Parkinson’s disease-like pathology in mice overexpressing mutated α-synuclein. Transl. Neurodegener. 8:7. 10.1186/s40035-019-0147-y 30867902 PMC6396507

