# Supplementary material for: Aquaporin-4 Polymorphisms Are Associated With Cognitive Performance in Parkinson’s Disease
Source: Front Aging Neurosci. 2022 Mar 9;13:740491. doi: 10.3389/fnagi.2021.740491 (PMC8959914; doi:10.3389/fnagi.2021.740491)
Supplement: Supplementary file 1 [file Table_1.DOCX]

**
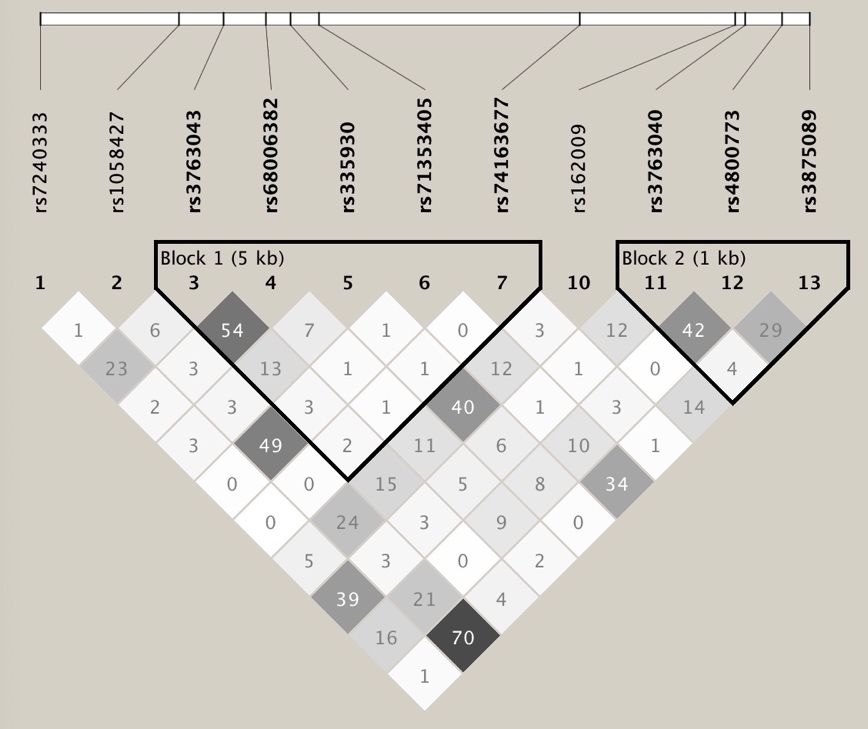
**

**Supplementary Figure 1. Linkage disequilibrium plot of investigated *AQP4* SNPs**

**Supplementary Table 1a. The association between investigated *AQP4* SNPs and *APOE* ε4**

|  | *APOE* ε4 carrier in *AQP4* non-minor allele carrier | *APOE* ε4 carrier in *AQP4* minor allele carrier | Pearson Chi-square value | P Value |
| --- | --- | --- | --- | --- |
| rs7240333_T | 66/301 | 25/81 | 2.809 | 0.094 |
| rs3763043_T | 43/179 | 48/203 | 0.007 | 0.931 |
| rs68006382_G | 63/255 | 28/127 | 0.330 | 0.566 |
| rs3763040_A | 47/230 | 44/152 | 3.654 | 0.056 |
| rs162009_A | 37/168 | 54/214 | 0.534 | 0.465 |

**Supplementary Table 1b. The association between investigated *AQP4* SNPs and *COMT* Val^158^Met**

|  | *COMT* GG | | *COMT* AG | | *COMT* AA | | Pearson Chi-square value | P Value |
| --- | --- | --- | --- | --- | --- | --- | --- | --- |
|  | non-minor allele carrier | Minor allele carrier | non minor allele carrier | Minor allele carrier | non minor allele carrier | Minor allele carrier |  |  |
| rs7240333_T | 80/301 | 25/81 | 142/301 | 32/81 | 79/301 | 24/81 | 1.522 | 0.467 |
| rs3763043_T | 39/179 | 66/203 | 93/179 | 81/203 | 47/179 | 56/203 | 7.077 | **0.029** |
| rs68006382_G | 64/255 | 41/127 | 123/255 | 51/127 | 68/255 | 35/127 | 2.832 | 0.243 |
| rs3763040_A | 68/230 | 37/152 | 107/230 | 67/152 | 55/230 | 48/152 | 3.023 | 0.221 |
| rs162009_A | 54/168 | 51/214 | 69/168 | 105/214 | 45/168 | 58/214 | 3.689 | 0.158 |

**Supplementary Table 1c. The association between investigated AQP4 SNPs and *GBA* variant carriers**

|  | *GBA* variant carrier in *AQP4* non-minor allele carrier | *GBA* variant carrier in *AQP4* minor allele carrier | Pearson Chi-square value | P Value |
| --- | --- | --- | --- | --- |
| rs7240333_T | 38/301 | 13/81 | 0.647 | 0.421 |
| rs3763043_T | 24/179 | 27/203 | 0.001 | 0.975 |
| rs68006382_G | 34/255 | 17/127 | 0.000 | 0.989 |
| rs3763040_A | 31/230 | 20/152 | 0.008 | 0.928 |
| rs162009_A | 21/168 | 30/214 | 0.188 | 0.665 |

*GBA* variants observed include A456P, E326K, G115R/G193E, G2019S/84GG, I489L, IVS2+1G>A, K(-27)R, L444P, N370S, R39C, R44C, R463C, T257I, T297S, T369M, T369M/R120W
